# New DVD supports trachoma surgery training

**Published:** 2011-12

**Authors:** Saul N Rajak, Amir Bedri, Matthew J Burton

**Affiliations:** International Centre for Eye Health, London School of Hygiene and Tropical Medicine, Keppel Street, WC1E 7HT, UK; Light For The World, Addis Ababa, Ethiopia; International Centre for Eye Health

**Figure:**
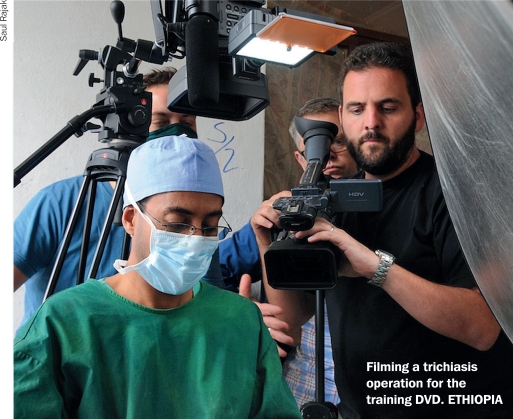


Trachomatous trichiasis (TT) is the blinding consequence of infective trachoma. It occurs when in-turned eyelashes scrape the cornea, and it affects over eight million people worldwide. It is painful and has been likened to thorns scraping your eyes every time you blink. Lid rotation surgery, which restores the in-turned eyelashes to the proper position, is the mainstay of treatment for TT. Unfortunately, the results of surgery can be poor with many patients developing recurrent trichiasis. There is evidence to suggest that poor surgical technique is responsible for a significant proportion of the recurrent cases. There is a pressing need to strengthen surgical training.

To help strengthen trichiasis surgery programmes, the International Centre for Eye Health (ICEH) has produced a comprehensive TT surgery training DVD, which was filmed in Ethiopia.

Currently, trainee trichiasis surgeons undertake a course of about two weeks, containing both theoretical and practical components. Training programmes usually teach one of the WHO approved lid rotation procedures: bilamellar tarsal rotation (BLTR) or posterior lamellar tarsal rotation (PLTR). These are described in the WHO trichiasis surgery training and certification manuals. A frequent limitation of training is the lack of exposure to surgical cases. Moreover, many trained surgeons do not operate frequently and most do not receive regular supervision.

The ICEH DVD contains step-by-step teaching videos of both BLTR and PLTR procedures. In addition, there is extensive supporting material, such as the assessment and counselling of patients, setting up an operating theatre, sterilising instruments and post-operative care.

The DVD will be distributed **free of charge**, in bulk, to National Trachoma Control programmes and non-governmental organisations for free distribution in training programmes. In addition, it can be obtained free of charge from TALC (Email info@talcuk.org or see page 30).

It is available in English and French, and was made possible thanks to the support of Band Aid via Fight For Sight, the Carter Center, the International Trachoma Initiative, and Stanton Media.

For this issue on instruments and consumables, we have specially adapted the following extract from the DVD. It covers using a steam autoclave to sterilise the instruments used in trachoma surgery.

## Using a steam autoclave

**Figure:**
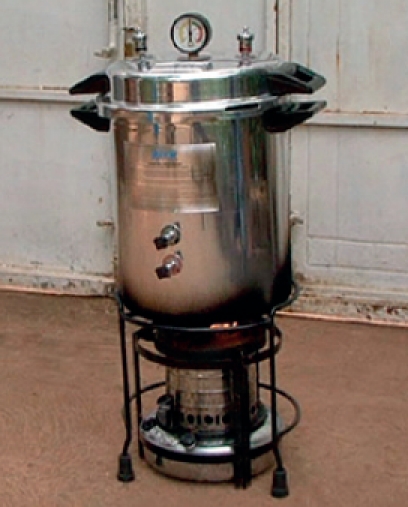


**Figure 1:**
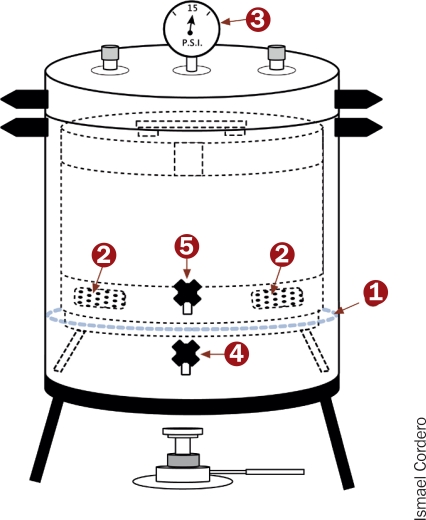
Diagram of a kerosene-burning steam-pressure autoclave

The kerosene-burning steam-pressure autoclave (Figure 1) is widely available in trachoma endemic regions. If you use a different model of autoclave you **must** read and carefully follow the instructions.

- You should use the autoclave in a clear area. Place the items to be sterilised on a clean table or shelf nearby.
- Pour water (ideally, distilled or demineralised water) into the autoclave up to the level of the top of the tripod stand inside (1).
- Load the drum with the items to be sterilised:
  — All jointed instruments should be placed in the opened or unlocked position.
  — Sharp edges must be protected by gauze or tubing to prevent dulling.
  — The drum should not be packed tightly.
    **Figure:**
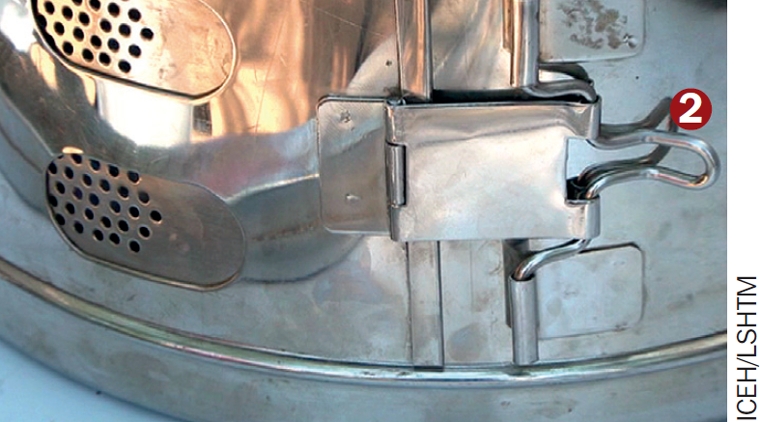
- You must check the drum to ensure that the vents are open. Close the drum lid and lock the vent collar in the open position (2).
- Place the drum in the autoclave and close the autoclave lid by rotating it clockwise, until it locks into position. You **must** ensure the autoclave lid is correctly closed. If this is not done properly, the autoclave is extremely dangerous when being heated.
- Check both taps are closed and place the autoclave on a kerosene burner. Some autoclaves are designed to be heated using an electrical supply.
  **Figure:**
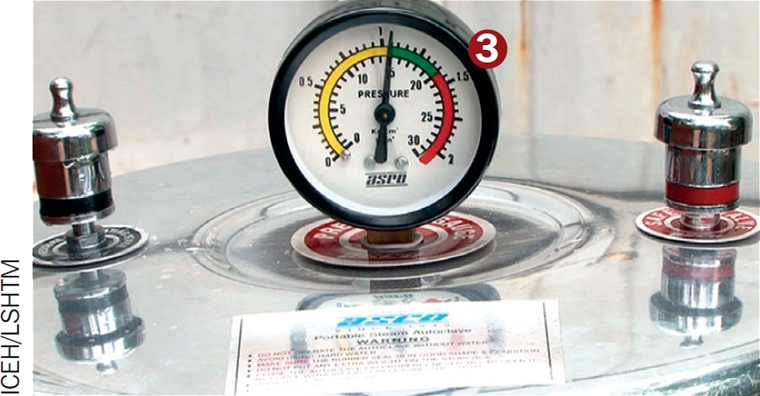
- After several minutes, the steam produced causes the pressure to rise in the autoclave. You can see this on the pressure gauge (3).
- When the pressure reaches 15 +/− 1 psi, steam will come out of the pressure valve. You must record the time at which this happens.
- Continue heating for a further 20–25 minutes. Turn off the heat source.
- Open the lower tap, which is the vacuum release tap. All the steam and water will then drain from the autoclave (4). As soon as the water stops draining, close the tap again and leave the autoclave for 5–10 minutes. A further vacuum develops in the autoclave, which will now dry the sterile contents.
- Then open the upper tap (5), which is the steam release tap, allowing all remaining steam to come out. The pressure gauge should now drop to zero psi.
- Only open the autoclave when the pressure is zero. Then remove the drum and immediately close the vents.

## Safety

There are some crucial safety points that you must always follow when using an autoclave.

- You should be well trained in autoclave usage and you should be trained and tested periodically for proficiency in the operation of an autoclave. There is a danger of transmitting HIV, hepatitis viruses, or other infectious diseases if the surgical materials are not properly sterilised. There is also a danger of causing serious injury if the autoclave is not used correctly.
- Always ensure there is the correct amount of water in the autoclave before every use.
- You must **not** open the autoclave until the pressure reaches zero.
- Do **not** use an autoclave if parts are malfunctioning; for example, if the pressure does not rise and there is continual escape of steam.
- Do **not** use if you notice wear and tear on the lid gasket or notice leaky taps and valves.

